# Micro- and Macroscale Assessment of Posterior Cruciate Ligament Functionality Based on Advanced MRI Techniques

**DOI:** 10.3390/diagnostics11101790

**Published:** 2021-09-28

**Authors:** Lena Marie Wilms, Karl Ludger Radke, Daniel Benjamin Abrar, David Latz, Justus Schock, Miriam Frenken, Joachim Windolf, Gerald Antoch, Timm Joachim Filler, Sven Nebelung

**Affiliations:** 1 Department of Diagnostic and Interventional Radiology, Medical Faculty, University Hospital of Dusseldorf, D-40225 Dusseldorf, Germany; Ludger.Radke@med.uni-duesseldorf.de (K.L.R.); DanielBenjamin.Abrar@med.uni-duesseldorf.de (D.B.A.); justus.schock@med.uni-duesseldorf.de (J.S.); Miriam.Frenken@med.uni-duesseldorf.de (M.F.); Antoch@med.uni-duesseldorf.de (G.A.); sven.nebelung@med.uni-duesseldorf.de (S.N.); 2 Department of Orthopedics and Trauma Surgery, Medical Faculty, University Hospital of Dusseldorf, D-40225 Dusseldorf, Germany; David.Latz@med.uni-duesseldorf.de (D.L.); joachim.windolf@med.uni-duesseldorf.de (J.W.); 3 Institute for Anatomy I, Heinrich-Heine-University, D-40225 Dusseldorf, Germany; timm.filler@uni-duesseldorf.de

**Keywords:** magnetic resonance imaging, posterior cruciate ligament, knee joint stability, loading, quantitative imaging, posterior drawer test

## Abstract

T2 mapping assesses tissue ultrastructure and composition, yet the association of imaging features and tissue functionality is oftentimes unclear. This study aimed to elucidate this association for the posterior cruciate ligament (PCL) across the micro- and macroscale and as a function of loading. Ten human cadaveric knee joints were imaged using a clinical 3.0T scanner and high-resolution morphologic and T2 mapping sequences. Emulating the posterior drawer test, the joints were imaged in the unloaded (δ_0_) and loaded (δ_1_) configurations. For the entire PCL, its subregions, and its osseous insertion sites, loading-induced changes were parameterized as summary statistics and texture variables, i.e., entropy, homogeneity, contrast, and variance. Histology confirmed structural integrity. Statistical analysis was based on parametric and non-parametric tests. Mean PCL length (37.8 ± 1.8 mm [δ_0_]; 44.0 ± 1.6 mm [δ_1_] [*p* < 0.01]), mean T2 (35.5 ± 2.0 ms [δ_0_]; 37.9 ± 1.3 ms [δ_1_] [*p* = 0.01]), and mean contrast values (4.0 ± 0.6 [δ_0_]; 4.9 ± 0.9 [δ_1_] [*p* = 0.01]) increased significantly under loading. Other texture features or ligamentous, osseous, and meniscal structures remained unaltered. Beyond providing normative T2 values across various scales and configurations, this study suggests that ligaments can be imaged morphologically and functionally based on joint loading and advanced MRI acquisition and post-processing techniques to assess ligament integrity and functionality in variable diagnostic contexts.

## 1. Introduction

Morphologic magnetic resonance imaging (MRI) is the most powerful contemporary imaging modality for the knee joint. Yet, morphologic MRI techniques are diagnostically limited in detecting and differentiating acute and chronic injuries of the posterior cruciate ligament (PCL). The acutely injured PCL may maintain continuity as a single structure, thereby misleading the reader to misclassify it as intact [1,2]. The chronically injured PCL may only demonstrate slight thickening and appear intact, too, while being functionally insufficient [3]. Furthermore, differentiating the chronically injured PCL versus the intact PCL is challenging because the PCL exhibits low signal intensity if scarred in elongation (and healed insufficiently) [2,4,5]. In such cases, quantitative MRI techniques such as T2 mapping may be diagnostically beneficial [6]. While for cartilage and meniscus, T2 mapping techniques have been used to quantify relevant ultrastructural and compositional tissue properties [7,8,9], literature data for the PCL are scarce. Wilson et al. reported that mean T2 values are higher in injured PCLs than in asymptomatic volunteers and that additional texture features are diagnostically beneficial [6]. Yet, this study lacked histologic correlation and functional evaluation of the PCL and had to rely on stress radiographs of the knee to establish PCL deficiency. For decades, stress radiography has been considered the gold standard to quantify PCL deficiency [10], confirming the diagnostic benefit of simultaneous stress application and imaging. Up to now, however, neither morphologic nor quantitative MRI techniques have been used to study the PCL and its appearance in response to loading. Anecdotical evidence suggests that the PCL is stretched out in high degrees of flexion [11], which may be used for diagnostic purposes [12].

Thus, the objective of this study was to bring together standardized joint loading, advanced MRI acquisition and post-processing techniques (in terms of T2 mapping and texture feature analyses), and macroscopic and microscopic cross-referencing to study the imaging appearance of the PCL in functional contexts and to define normative values of the intact PCL. Our hypothesis was that, under loading, the PCL and its osseous insertion sites exhibit significant changes in T2 relaxation as an indication of intact ligament function and stable load transmission.

## 2. Materials and Methods

### 2.1. Study Design

The present study was designed as an in situ imaging study on ten fresh frozen human knee joint cadaveric specimens obtained from body donors who had deceased owing to unrelated conditions. Written informed consent by the body donors was obtained before the study. Approval of the local institutional review board (Ethical Committee of the Medical Faculty of Heinrich-Heine University, Düsseldorf, Germany, 2019-682) was obtained prior to the study’s initiation and all relevant local guidelines and regulations were strictly obeyed.

### 2.2. Human Cadaveric Knee Joint Specimens

#### 2.2.1. Sample Size Estimation

Minimum sample size was estimated as eight based on the power analysis of the initial three specimens (power of 0.8, probability of type-I-error of 0.05, effect size of 1.6, two-tailed, www.statstodo.com, accessed on 12 May 2020).

#### 2.2.2. Pre-Measurement Specimen Preparations

The local Institute for Anatomy I (Heinrich-Heine University) provided ten fresh unfixed human knee joint specimens. Body donors’ mean age at death was 80.3 ± 7.9 years (range, 64–91 years) and we included three male and seven female specimens, of which four were right and six were left specimens. If frozen prior to the measurements, specimens were left to thaw at room temperature for at least 12 h. The tibiae were extended by a tapered polyvinyl-chloride medullary rod driven into the medullary cavity of the diaphyses and, subsequently, fixed using liquid polymethyl-methacrylate (Technovit-3040, Heraeus-Kulzer, Wehrheim, Germany).

### 2.3. Loading Device

**Figure 1:**
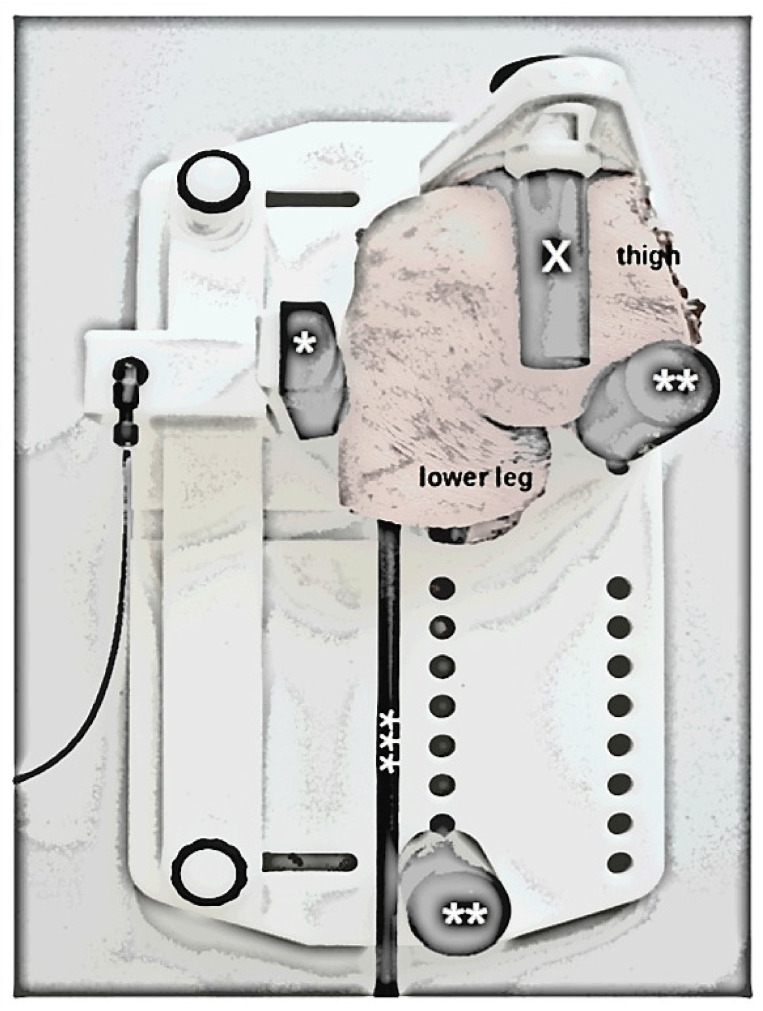
Experimental setup to image the functionality of the posterior cruciate ligament in human knee joint specimens. The specimen was laterally positioned in 90° of flexion. The thigh was fixed in a knee-holding apparatus (X) to prevent displacement under loading. Padded pressure applicator below the tibial tuberosity (*), padded abutments as counter-bearings (**), and polyvinyl-chloride rod in the medullary cavity of the tibia (***). Sketched visualization of the specimen-loaded device. Same right knee joint specimen as in Figure 2 and Figure 4.

Loading was realized using a commercial MRI-compatible pressure-controlled loading device (Telos Stress Device Type SE-MR, Telos GmbH, Wölfersheim, Germany), which had been validated before [13]. In contrast to the earlier validation study that had been conducted on human ankle joints, the present study used a different loading setup to emulate the posterior drawer test by pressure-controlled posterior displacement of the tibia relative to the fixed femur (Figure 1). In line with the manufacturer’s instructions, a dedicated knee holding apparatus that was freely adjustable to the joint’s anatomy was used to fix the specimens in a lateral position of 90° flexion. The padded pressure applicator was placed loosely below the tibial tuberosity and was actuated by the pneumatic mechanism via set pressure levels. When pressurized, the padded pressure applicator effected direct loading via posterior translation of the tibia. Padded counter-bearings at the distal dorsal upper leg and the extended lower leg were used for additional fixation. In patients, the second counter-bearing would be in contact with the distal dorsal lower leg, whereas in our setup, the knee joint-only specimens had to be extended distally to compensate for the lacking below-knee extremity. The pneumatic mechanism was connected to the control unit located outside of the MRI room. Connected to the loading unit via standard pressure tubes, the control unit was pressurized by disposable carbon dioxide cartridges (16 g at 50.9 bar equaling an expanded volume of 8.7 l [15 °C], Telos GmbH, Wölfersheim, Germany). Pressure levels were set by control of the manometer.

### 2.4. Image Acquisition and Analysis

#### 2.4.1. MR Image Acquisition

A clinical 3.0T MRI system (Magnetom Prisma, Siemens Healthineers, Erlangen, Germany) was used for imaging. All specimens were positioned in the loading device as detailed above and imaged in the unloaded (δ_0_) and loaded configurations (δ_1_) using a flexible 18-channel body coil (Siemens Healthineers) that fully covered the specimen and device. Following imaging in the δ_0_-configuration, pressure was set to 2.3 bar, resulting in effective posterior forces on the tibia of 147 N (=15 kP), per validation of the manufacturer. After 5 min of equilibration following the initiation of loading, the joint was imaged in the δ_1_-configuration. For each joint and configuration, the imaging protocol consisting of morphologic and quantitative sequences was completed. For morphologic reference imaging, a 3D proton density weighted fat-saturated turbo spin-echo sequence commercially available as the SPACE sequence (Sampling Perfection with Application optimized Contrasts using different flip angle Evolutions, Siemens Healthineers) was used. For quantitative T2 mapping, a multi spin-echo sequence was obtained. While the SPACE sequence was acquired in the sagittal orientation, the T2 mapping sequence was acquired in the parasagittal orientation, i.e., aligned along the course of the PCL. The sequence details are indicated in Table 1. Constant joint flexion of 90° was confirmed using the morphologic sequences at the δ_0_- and δ_1_-configurations. Morphologic signs indicative of previous PCL injury such as gross fiber discontinuity, abnormal diameter or configuration of the PCL, and an excessively posterior position of the tibia relative to the femur [6,14] were assessed during image acquisition and found to be absent in all specimens. Consequently, no specimen had to be excluded. Imaging was performed at room temperature.

#### 2.4.2. Manual Segmentations and Image Post-Processing

Manual segmentations were performed by L.M.W. (clinical radiologist with 5 years of experience in musculoskeletal imaging), who delineated each structure using the polygon mode and brush tool of ITK-SNAP software (v3.8, Cognitica, Philadelphia, PA, USA) [15]. For each joint and configuration, all parasagittal SPACE slices that visualized the PCL without interference of partial volume effects because of synovial fluid were included in the segmentation outlines. Consequently, on average, 3.0 ± 0.7 parasagittal slices were analysed per joint and configuration. Customized routines implemented in Matlab (R2018b; Natick, MA, USA) were used to automatically determine the length of the PCL based on the segmentation outlines. To this end, a 3D center line was calculated along the course of the PCL and used to quantify the length of the PCL from the proximal to the distal insertion site.

**Figure 2:**
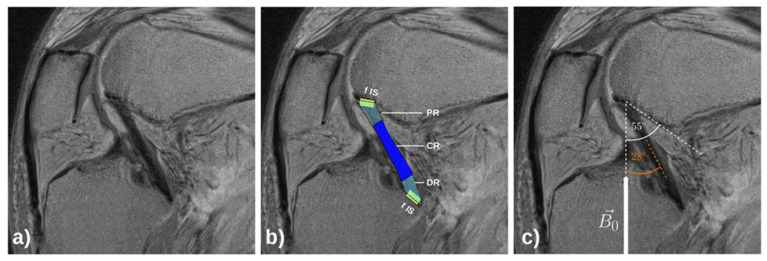
Exemplary visualization of the osseous and ligamentous regions-of-interest of the posterior cruciate ligament (PCL) and the orientation of the PCL to the main magnetic field B_0_. On the corresponding morphologic parasagittal image with moderate T2-weighting (echo time = 41.4 ms) (**a**), the PCL was identified and manually segmented. The PCL’s segmentation outline was automatically divided into quarters for further subregional analysis (**b**). The proximal 25% of the PCL was defined as the proximal subregion (PR, light blue), the central 50% as the central subregion (CR, blue), and the distal 25% as the distal subregion (DR, light blue). The femoral (fIS) and tibial insertion sites (tIS) of the PCL were positioned around the cortical bone of the respective insertion sites and are outlined by rectangular boxes (yellow). The white arrow indicates the orientation of the main magnetic field B_0_ (**c**). The orange dashed lines indicates the principal orientation of the PCL and an angle of 28° (orange) was determined between the PCL and the main magnetic field. Similarly, the orientation of the magic angle of 55° with respect to B_0_ is indicated by the white dashed lines. Same right knee joint specimen as in Figure 1 and Figure 4 (δ_1_-configuration).

To determine regional differences within the PCL, the ligament was automatically divided into three subregions along its entire length as additional regions-of-interest (ROIs), i.e., the proximal region (PR, proximal 25% of the ligament’s length originating at the femoral insertion), the central region (CR, central 50% of the ligament’s length), and the distal region (DR, distal 25% of the ligament’s length extending into the tibial insertion). Furthermore, the PCL’s osseous insertion sites at the femur and tibia were defined as additional ROIs. Using customized routines implemented in Python software (v3.7.3; Beaverton, OR, USA, https://www.python.org/, accessed on 28 June 2020), standardized rectangular boxes of 0.5 × 1.0 cm^2^ [height × width] were centered around the proximal and distal edges of the PCL’s segmentation outlines on the central parasagittal slice. To include the different constituents of the PCL footprint, i.e., ligament, (un)calcified fibrocartilage, and bone, the boxes were automatically positioned parallel to the footprint’s cortical bone interface, with a quarter of the box oriented to the bone and three quarters of the box oriented to the PCL. Figure 2 gives an example of the ligamentous and osseous ROIs of the PCL.

Additionally, the anterior cruciate ligament (ACL) and the anterior and posterior horns of the medial and lateral menisci were segmented on SPACE images oriented strictly sagittal. On average, these structures were segmented in 2.2 ± 0.9 (ACL) and 4.7 ± 1.4 (menisci) images.

Following their delineation on the SPACE sequence, each structure’s segmentation outlines were visually checked to eliminate potential partial volume effects, so that only pixels that safely belonged to the ligament or meniscus were included and quantified. The segmentation outlines were then transferred to the corresponding T2 maps, which were generated using Python software and customized routines. To this end, non-linear least-square fits were applied to the exponential decay curves in a pixel-wise manner. R^2^ statistics adjusted to the degrees of freedom were used to verify fit quality and only pixels with R^2^-values ≥0.9 were included.

Overall, T2 characteristics were determined for each joint, configuration, and ROI.

#### 2.4.3. Texture Feature Analysis

Texture feature analysis may support interpretation of image data by providing information not readily appreciable by the human eye [16]. Grey-level co-occurrence matrices (GLCMs) were used to quantify the texture features entropy, variance, contrast, and homogeneity to characterize ligament functionality beyond mere summary statistics [7,17,18]. Practically, these features were determined on the PCL’s segmentation outlines and T2 maps based on four orientations, i.e., 0°, 45°, 90°, and 135°, using an offset of a single pixel [7,17,19]. The routine was implemented in Matlab and texture features were determined for each joint and configuration for the entire PCL and each subregion.

### 2.5. Macroscopic and Microscopic Reference Analysis

Immediately after imaging, the specimens were prepared for macroscopic and microscopic reference evaluation of the PCL and its osseous insertions sites [20,21,22]. Following surgical access to the joint via the medial parapatellar approach and lateral eversion of the patella, the cruciate ligament complex was identified and evaluated macroscopically. Using an electric saw and a rongeur, the medial femoral condyle and the posterocentral tibial plateau were sampled and cut to standard dimensions of 2 × 2 × 2 cm^3^. Condyle and plateau were sectioned through the centers of the femoral and tibial PCL insertion sites along the parasagittal orientation. Consequently, the femoral and tibial insertion sites and the attached proximal and distal stumps of the PCL were harvested for microscopic analysis. Samples were fixed and decalcified in Ossa fixona (Diagonal, Münster, Germany), dehydrated, embedded in paraffin, and cut to 5 μm thick slices. Staining with hematoxylin/eosin, Safranin O, and Picrosirius red was performed in line with established protocols [23,24,25]. A conventional light microscope (Motic Easy Scan Infinity 100, MoticEurope, Barcelona, Spain) and dedicated software (Motic^®®^ Images Devices MoticEurope, Barcelona, Spain) were used for image documentation and analysis.

The macroscopic and microscopic appearances of the PCL were graded as normal (i.e., no changes), abnormal (i.e., thinned or thickened without fiber disruption or displaying signs of degeneration such as sclerosis or mucoid transformation), or ruptured (i.e., thinned or thickened with partial or complete fiber disruption) [20,22]. L.M.W. and T.F. (anatomist with 33 years of experience in musculoskeletal anatomy) performed the reference readings in consensus.

### 2.6. Statistical Analysis

Statistical analysis was performed by the first author (L.M.W.) using GraphPad Prism (v5.0, GraphPad Software, San Diego, CA, USA). Assuming underlying normal distribution, PCL lengths were assessed as a function of loading and compared using the two-tailed paired Student’s *t*-test. Not assuming normal distributions for T2 relaxation times and associated texture features, non-parametric tests were used to compare two groups (i.e., the Wilcoxon signed rank test) or more than two groups (i.e., Friedman’s test), respectively, followed by Dunn’s multiple comparison test wherever appropriate. To contain the number of statistically significant, yet clinically (most likely) insignificant findings, the level of significance was set to *p* ≤ 0.01. Unless indicated otherwise, data are presented as mean ± standard deviation.

## 3. Results

**Figure 3:**
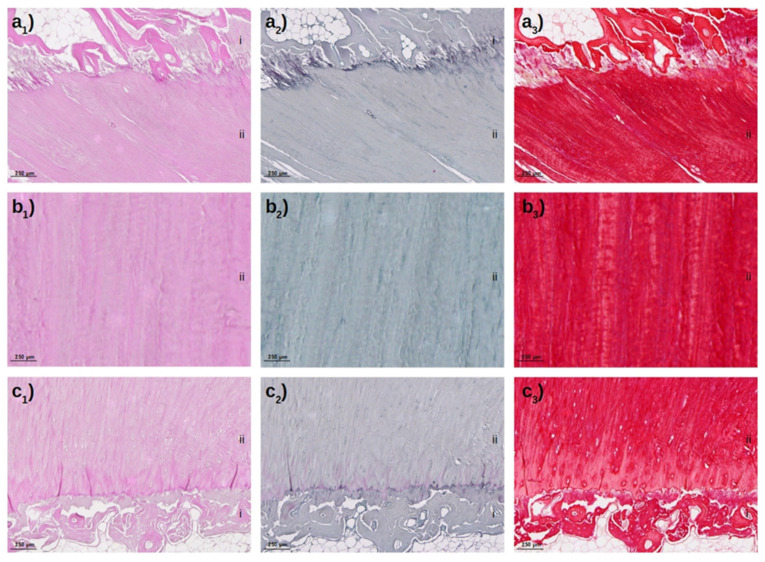
Microscopic reference evaluation of the posterior cruciate ligament (PCL). Displayed are close-up views of the femoral (**a_1_**–**a_3_**) insertion sites, the ligamentous portion (**b_1_**–**b_3_**), and the tibial insertion site (**c_1_**–**c_3_**) of a representative PCL following hematoxylin and eosin (**a_1_**,**b_1_**,**c_1_**), Safranin O (**a_2_**,**b_2_**,**c_2_**), and Picrosirius red staining (**a_3_**,**b_3_**,**c_3_**). The PCL insertion sites are intact with regular bone and calcified fibrocartilage (i) and ligament (ii). In this specimen and throughout, fibers were oriented parallel without any fiber discontinuity. Other magnifications indicated the absence of relevant thickening or thinning and degenerative changes (not shown). Bars correspond to 250 µm.

All ten specimens underwent complete MR imaging and subsequent macro- and microscopic reference evaluation. The macroscopic appearance of the PCL was normal in all ten knee joints. Microscopically, all PCLs were normal, too, without alterations of ligament size, disruptions of fiber continuity, or degenerative changes (Figure 3).

**Figure 4:**
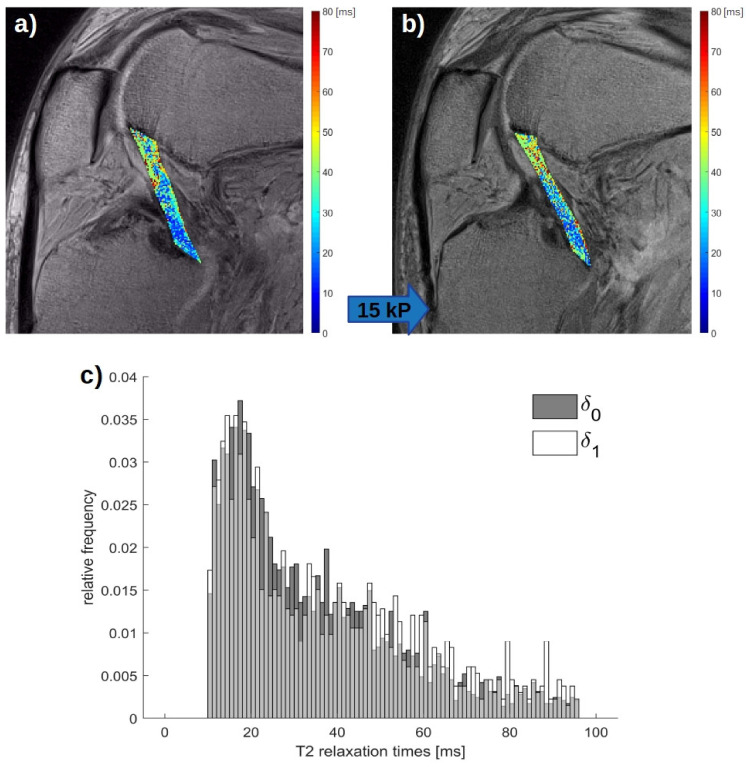
Quantitative T2 maps in the unloaded and loaded configurations and associated histogram of the posterior cruciate ligament (PCL). In this representative knee joint, spatially resolved T2 maps are displayed in the unloaded (**a**), (δ_0_) and loaded configurations (**b**), (δ_1_). T2 maps of the segmented PCL outlines were overlaid onto the corresponding T2-weighted morphologic images (echo time = 41.4 ms). The blue block arrow indicates the direction and magnitude of loading. T2 relaxation times are color-coded and range from 0 to 80 ms (right). (**c**) The associated histogram of pixel-wise T2 relaxation times of the PCL in this specimen indicates relative increases of high-T2 pixels in the δ_1_-configuration (white) as compared with more prevalent low-T2 pixels in the δ_0_-configuration (dark grey). The light grey bars indicate the overlaying T2 pixels of the δ_0_-configuration and δ_1_-configuration. Same right knee joint specimen as in Figure 1 and Figure 2.

Morphologically, the PCL was more stretched and flattened when loaded. Consequently, loading induced significant changes in the mean length of the PCL (δ_0_: 37.8 ± 1.8 mm; δ_1_: 44.0 ± 1.6 mm; *p* ≤ 0.01). In the T2 maps, the PCL underwent the most distinct changes in its proximal region, where low signal areas disappeared, while loading induced changes in the central and distal regions were less pronounced (Figure 4a,b). Correspondingly, T2 relaxation times shifted more to higher values under loading (Figure 4c).

Quantitatively, summary statistics and texture variables of the T2 maps indicated variable changes as a function of loading and ROI (Table 2). For the entire PCL, significant increases in response to loading were found for mean T2 values (δ_0_: 35.5 ± 2.0 ms; δ_1_: 37.9 ± 1.3 ms; *p* = 0.01). Besides, mean T2 values increased in all subregions, most prominently in the PR, even though non-significantly. ROI-wise comparisons revealed significant differences between the subregions in the δ_1_-configuration (*p* < 0.01) that were not present in the δ_0_-configuration (*p* = 0.14). Post-hoc testing revealed these differences to be due to the significant difference between PR and DR. For entropy, significantly different values were found within the PCL, at both δ_0_ and δ_1_, with the CR displaying significantly larger entropy values than the DR, irrespective of loading (*p* ≤ 0.01). For contrast, loading induced significant increases in the entire PCL (δ_0_: 4.0 ± 0.6; δ_1_: 4.9 ± 0.9; *p* = 0.01), in the PR (*p* < 0.01), and in the CR (*p* = 0.01). Otherwise, post-hoc differences of texture features in the distinct ROIs revealed no significant differences for contrast, homogeneity, or variance.

Response-to-loading patterns of the osseous insertion sites, the ACL, and medial and lateral menisci were characterized by variable, though non-significant changes in the T2 maps under loading (Table 3). Supplementary Video S1 visualizes the T2 values of the medial and lateral meniscus as well as the ACL of a representative knee joint.

## 4. Discussion

The most important finding of this study is that functional imaging of the PCL and its osseous insertion sites based on T2 mapping, texture feature analysis, and standardized loading is feasible and may be used to quantify ligament functionality beyond mere structure and morphology. With regards to prospective diagnostic utilization, this study provides normative values of T2 relaxation times and associated texture features of the (macro- and microscopically) intact PCL in different functional configurations.

In our study, the length of the unloaded PCL at 90° flexion was 37.8 ± 1.8 mm, which is well in line with earlier literature data [26]. Under loading, its length increased significantly by an average of 6.2 mm, thereby demonstrating the loading mechanism’s efficiency in stressing the PCL alongside other passive stabilizers. Our results also indicate a substantial physiological laxity of the PCL at 90° flexion, which, to the best of our knowledge, has not been reported before, as previous studies tended to be focused on loading induced changes of the joint, not the PCL.

This study provides normative values for T2 and the underlying texture parameters as a function of loading, for both the PCL and other intra-articular structures. At δ_0_, we found mean T2 relaxation times of 35.2 ± 2.0 ms for the entire PCL, which is well in line with earlier literature data, too [6,7]. Quantifying the PCL’s T2 relaxation times of asymptomatic volunteers, Wilson et al. reported mean values of 29–37 ms, depending on the subregion [7]. Upon loading, we found increases in T2 that were significant for the entire PCL and tended towards significance in the distinct subregions. Potential contributing factors involve compositional and (ultra-)structural changes of the PCL under loading. Similar to other ligaments, the PCL consists primarily of water (~70% of wet weight) and type-I collagen (~20%), while other collagens (~3–5%), elastin (~1–2%), glycoproteins (~1–2%), and proteoglycans (<1%) contribute to lesser extents [27]. For articular cartilage, the association of quantitative MRI parameters and distinct tissue properties has been clarified and the correspondence of T2 and tissue hydration has been firmly established [8,28,29,30,31]. Because significant loading induced alterations of PCL composition, let alone increases in its water content, are unlikely, ultrastructural changes provide the most likely explanation of these findings. Under loading, the material behavior across different physical scales, i.e., from the collagen molecule to the collagen fibril and fascicle to the ligament, is complex and, in large parts, still poorly understood [32]. It is clear, however, that loading is characterized by non-linear anisotropic force-displacement and stress–strain relationships. In biomechanical contexts, this translates to initial stretching to the contour length (on the molecular level) and uncrimping and straightening (on the fibrillar level) at low strains, manifesting as a soft toe region in the stress–strain curves (≤2% strain). If loaded further (>2% strain), a stiff linear region of elastic elongation follows. Once all collagen fibrils and fascicles are uncrimped, the collagen fibers stretch, and the ligament deforms linearly owing to the inter-molecular sliding of the collagen molecules. In normal activity, most ligaments work in the toe and lower linear regions. In our setup, the exact strains on the PCL remain unclear, yet literature data indicate these forces to be substantial. Using a multiaxial robotic system, Fox et al., demonstrated in situ forces on the PCL of 112 ± 29 N at 90° of flexion [33]. Consequently, the PCL is considerably straightened and flattened across the different physical scales and in a more orderly configuration [34,35], which was confirmed by our study. Ligament straightening potentially alters the percentage of fibers oriented at the magic angle of 55° to the main magnetic field B_0_. On the tissue scale, the proximal part of the PCL is particularly prone to the magic angle effect because of its physiologic curvature. In structures with tightly bound collagen molecules, this effect results in artefactual signal increases owing to complex quantum mechanical associations. For the PCL, Gatehouse et al. found an increased signal in the PCL’s femoral portion, which is often oriented at the magic angle [36]. In line with these observations, we observed significantly higher mean T2 relaxation times (at δ_1_) for the proximal versus the distal region. Even though plausible, these findings are contradictory to earlier findings by Wilson et al., who found the highest T2 relaxation times in the distal (and not proximal) portion of the PCL [7]. Possible reasons for these discrepancies involve differences in the measurement configuration and imaging setup, MRI protocol, segmentation methodology, and ROI definition. Wilson et al. studied the knee joints using a dedicated knee coil [7], which implies a slightly flexed position of ~20°. Our loading device rendered the use of a dedicated knee coil impossible, and we had to resort to using a flexible multi-channel body coil. Biomechanically, assessing knee joint laxity at 90° of flexion is beneficial because it is at larger flexion that isolated PCL ruptures have their greatest effects [37]. Even though the first anecdotical evidence of a clinical benefit by flexing the knee to 90° has emerged [12], the exact effects of increasing joint flexion on T2 characteristics of the PCL (and the magic angle effect) need to be determined. In addition, cadaveric in situ studies (as in the present study) differ from clinical in vivo studies [6,7], because pain, muscle tension, and functional deficits cannot be assessed in situ. While in situ studies may only assess passive stabilizers of the knee, in vivo studies evaluate the contributions of active and passive stabilizers. Absolute T2 quantification is only valid within a particular setup, thereby limiting inter-study comparability.

Texture features may assess the spatial distributions and underlying tissue structure more comprehensively than mere summary statistics [17]. We found significant intra-ligamentous differences for entropy, irrespective of loading, and significant loading induced increases in contrast throughout the entire PCL (except for the DR). Entropy provides a measure of disorder in the pixel intensities with high entropy values indicating complex and disorganized texture. Thus, entropy can be used to detect changes in mostly homogeneous tissue areas. Our data indicate that the PCL is per se a variable structure and that loading does not decrease the PCL’s inherent state of disorganization. Contrast provides a measure of local variation of neighboring pixel values and, consequently, high contrast values suggest the presence of largely different and neighboring pixel intensities. Significant increases in contrast under loading may be the result of concurring changes within the ligament on the compositional and (ultra)structural level as indicated above, which increase tissue complexity by inducing simultaneous adaptive processes that increase and decrease T2 relaxation times alike.

T2 relaxation times of the PCL’s osseous insertion sites, the ACL, and the menisci did not change significantly under loading. Previous studies have demonstrated relatively low sensitivity of T2 in the assessment of altered biomechanics [38,39]. Hence, alternative techniques with different sensitivities such as UTE (ultrashort echo time)-T2* sequences for the PCL insertion [40], T1ρ for meniscus functionality [38], or diffusion-weighted imaging for the ACL [41] could be used in future studies.

This study has several limitations. First, owing to their advanced age, our specimens are not representative of the significantly younger clinical population. Second, owing to its laboratory design, the findings of our study are not directly transferrable to the clinical setting. Third, the exact correlate of T2 remains unclear even though sensitivity towards collagen fiber orientation and alignment as well as collagen, proteoglycan, and water contents has been proven for cartilage [8,42]. Additional reference studies using biochemical referencing [43] may help reveal the underlying adaptive processes even further. Fourth, we only evaluated the proximal and distal PCL portions and not the PCL in its entirety to reduce processing efforts. Consequently, the microscopic evaluation of PCL integrity had to be complemented by the macroscopic evaluation of the central ligament. Fifth, additional quantitative MRI parameters such as UTE-T2*, T1ρ, and others may offer additional insights into PCL functionality. However, in the context of biomechanical imaging, the additional time demand of the quantitative sequences (that necessarily have to be acquired in the unloaded and loaded configurations) must be balanced against the additional diagnostic benefit. Clinical usability needs to be defined for the underlying clinical questions and necessarily requires larger clinical studies. Against this background, once the device is operated in clinical contexts, related aspects of patient comfort and compliance, device handling and safety, and measurement reproducibility, accuracy, and validity may be elucidated, too.

## 5. Conclusions

If complemented by loading, T2 mapping and texture feature analysis quantify loading induced changes of the PCL. Its direct visualization and quantification on a compositional and (ultra)structural level help exploit the technique’s diagnostic capabilities in more functional contexts. By defining normative quantitative values for PCL length, T2 relaxation times, and associated texture features as a function of loading, this study provides an imaging framework for the simultaneous assessment of ligament integrity and functionality with potential applications in the future grading of PCL injuries, guiding of treatment, and monitoring of ligament healing.

## Figures and Tables

**Table 1:** Acquisition parameters of the MRI sequences.

	PDw fs	T2 Mapping
Acronym	SPACE	n/a
Sequence type	3D TSE	2D MSE
Orientation	sag	parasag (*)
Repetition time [ms]	1200	1530
Echo time [ms]	28	13.8/27.6/41.4/55.2/69.0
Turbo spin-echo factor	53	n/a
Field of view [mm]	160 × 160	160 × 160
Acquisition matrix [pixels]	256 × 256	384 × 384
Pixel size [mm/pixel]	0.6 × 0.6	0.4 × 0.4
Number of signal averages [n]	1	1
Slices [n]	192	20
Slice thickness/gap [mm]	0.5/0	3.0/0.2
Duration [min:sec]	20:16	09:28

PDw—proton density weighted; fs—fat-saturated; SPACE—Sampling Perfection with Application optimized Contrasts using different flip angle Evolutions, TSE—turbo spin-echo; MSE—multi spin-echo, (para)sag—(para)sagittal; n/a—not available. (*) aligned along the course of the PCL.

**Table 2:** Non-parametrical summary statistics and texture variables of T2 maps of the posterior cruciate ligament (PCL) and its subregions as a function of loading (δ_0_, δ_1_). Mean T2 relaxation times and standard deviations (SD) as well as texture variables, i.e., entropy, contrast, homogeneity, and variance, were determined for the entire PCL, the proximal subregion (PR), the central subregion (CR), and the distal subregion (DR). Unloaded (δ_0_) and loaded (δ_1_) configurations. Mean ± standard deviation. Median values are indicated in parentheses. For each region-of-interest and T2 parameter, δ_0_ and δ_1_ were compared using the Wilcoxon signed rank test and the respective *p*-values are displayed in columns (†). For each configuration and T2 parameter, the subregions were compared using the Friedman test followed by Dunn’s multiple comparison test. The respective *p*-values are displayed in rows (‡). Significant differences are indicated by ** or displayed in bold type.

T2		PCL				*p*-Values (‡)			
		Entire	PR	CR	DR	Overall	PR vs. CR	PR vs. DR	CR vs. DR
Mean	δ_0_	35.5 ± 2.0 (35.5)	41.1 ± 1.7 (40.6)	34.7 ± 0.7 (34.2)	32.7 ± 1.5 (31.5)	0.135	ns	ns	ns
	δ_1_	37.9 ± 1.3 (38.2)	45.7 ± 1.3 (44.5)	36.5 ± 0.7 (36.2)	33.4 ± 1.5 (33.0)	**<0.001**	ns	**	ns
	*p*-values (†)	**0.012**	0.027	0.193	0.695				
									
SD	δ_0_	20.4 ± 0.4 (20.2)	25.7 ± 3.5 (22.4)	22.8 ± 6.5 (19.8)	20.8 ± 3.3 (19.4)	**0.006**	ns	ns	ns
	δ_1_	21.7 ± 1.4 (21.9)	26.5 ± 7.5 (22.5)	21.4 ± 1.5 (21.4)	21.6 ± 3.3 (21.4)	0.067	ns	ns	ns
	*p*-values (†)	0.049	0.922	0.846	0.492				
									
Entropy	δ_0_	5.9 ± 0.1 (5.9)	5.6 ± 0.4 (5.9)	5.8 ± 0.1 (5.7)	5.5 ± 0.2 (5.6)	**<0.001**	ns	ns	**
	δ_1_	6.0 ± 0.1	5.8 ± 0.1 (5.9)	5.8 ± 0.2 (5.7)	5.4 ± 0.2 (5.6)	**0.001**	ns	ns	**
	*p*-values (†)	0.020	0.106	0.683	0.322				
									
Contrast	δ_0_	4.0 ± 0.6 (4.1)	2.4 ± 1.2 (2.9)	2.8 ± 1.4 (3.4)	3.3 ± 1.0 (2.7)	0.187	ns	ns	ns
	δ_1_	4.9 ± 0.9 (4.8)	4.7 ± 1.7 (3.6)	4.3 ± 1.2 (4.1)	4.5 ± 1.5 (3.4)	0.316	ns	ns	ns
	*p*-values (†)	**0.010**	**0.004**	**0.010**	0.027				
									
Homogeneity	δ_0_	0.6 ± 0.0 (0.6)	0.7 ± 0.2 (0.6)	0.7 ± 0.1 (0.6)	0.6 ± 0.1 (0.6)	0.316	ns	ns	ns
	δ_1_	0.5 ± 0.0 (0.6)	0.6 ± 0.1 (0.6)	0.6 ± 0.1 (0.6)	0.6 ± 0.1 (0.6)	0.710	ns	ns	ns
	*p*-values (†)	0.037	0.322	0.020	0.065				
									
Variance	δ_0_	390.8 ± 39.0 (398.5)	614.4 ± 207.3 (470.9)	717.9 ± 943.8 (368.6)	409.8 ± 129.2 (362.3)	0.046	ns	ns	ns
	δ_1_	455.4 ± 56.9 (471.3)	790.0 ± 633.7 (446.0)	433.8 ± 65.19 (399.7)	446.4 ± 154.2 (413.7)	0.187	ns	ns	ns
	*p*-values (†)	0.020	0.922	1.000	0.700				

**Table 3:** T2 relaxation times of other intra-articular structures in response to loading. T2 relaxation times are indicated for the osseous insertion sites of the posterior cruciate ligaments (PCL), the anterior cruciate ligaments (ACL), and the anterior and posterior horns of the medial and lateral meniscus, in both the unloaded (δ_0_) and loaded (δ_1_) configurations. Mean ± standard deviations [ms]. Wilcoxon signed rank test was used to determine if configurations were significantly different.

Anatomical Structures		T2 Relaxation Times (ms)		
		δ_0_	δ_1_	*p*-Values
PCL Insertion Sites	Femoral	55.4 ± 8.8	55.7 ± 10.5	0.85
	Tibial	52.9 ± 8.1	57.8 ± 8.5	0.13
ACL		46.2 ± 8.6	45.8 ± 10.7	0.77
Menisci	Anterior medial	34.4 ± 4.4	33.5 ± 5.3	0.85
	Posterior medial	29.7 ± 6.5	32.7 ± 7.5	0.04
	Anterior lateral	33.1 ± 3.3	32.3 ± 3.4	0.32
	Posterior lateral	32.4 ± 3.4	33.3 ± 4.3	0.49

## Data Availability

The data presented and/or analyzed in this study are available upon reasonable request from the corresponding author.

## Supplementary Materials

The following are available online at https://www.mdpi.com/article/10.3390/diagnostics11101790/s1, Supplementary Video S1: Quantitative T2 values of the anterior cruciate ligament (ACL) and the medial and lateral menisci in the unloaded and loaded configurations. In this representative knee joint, spatially resolved T2 maps are displayed in the obliquely acquired sequences of the unloaded (**a**) and loaded configurations (**b**). Following manual segmentations, the T2 maps of the ACL and meniscus outlines were overlaid onto the corresponding T2-weighted morphologic images (with an echo time of 41.4 ms). The blue block arrow indicates the direction and magnitude of loading. T2 relaxation times are color-coded and their scale ranges from 0 to 80 ms (below). Same right knee joint specimen as in Figure 1, Figure 2 and Figure 4.
