# Supplementary figures and images for: Micro- and Macroscale Assessment of Posterior Cruciate Ligament Functionality Based on Advanced MRI Techniques
Source: Diagnostics (Basel). 2021 Sep 28;11(10):1790. doi: 10.3390/diagnostics11101790 (PMC8535058; doi:10.3390/diagnostics11101790)

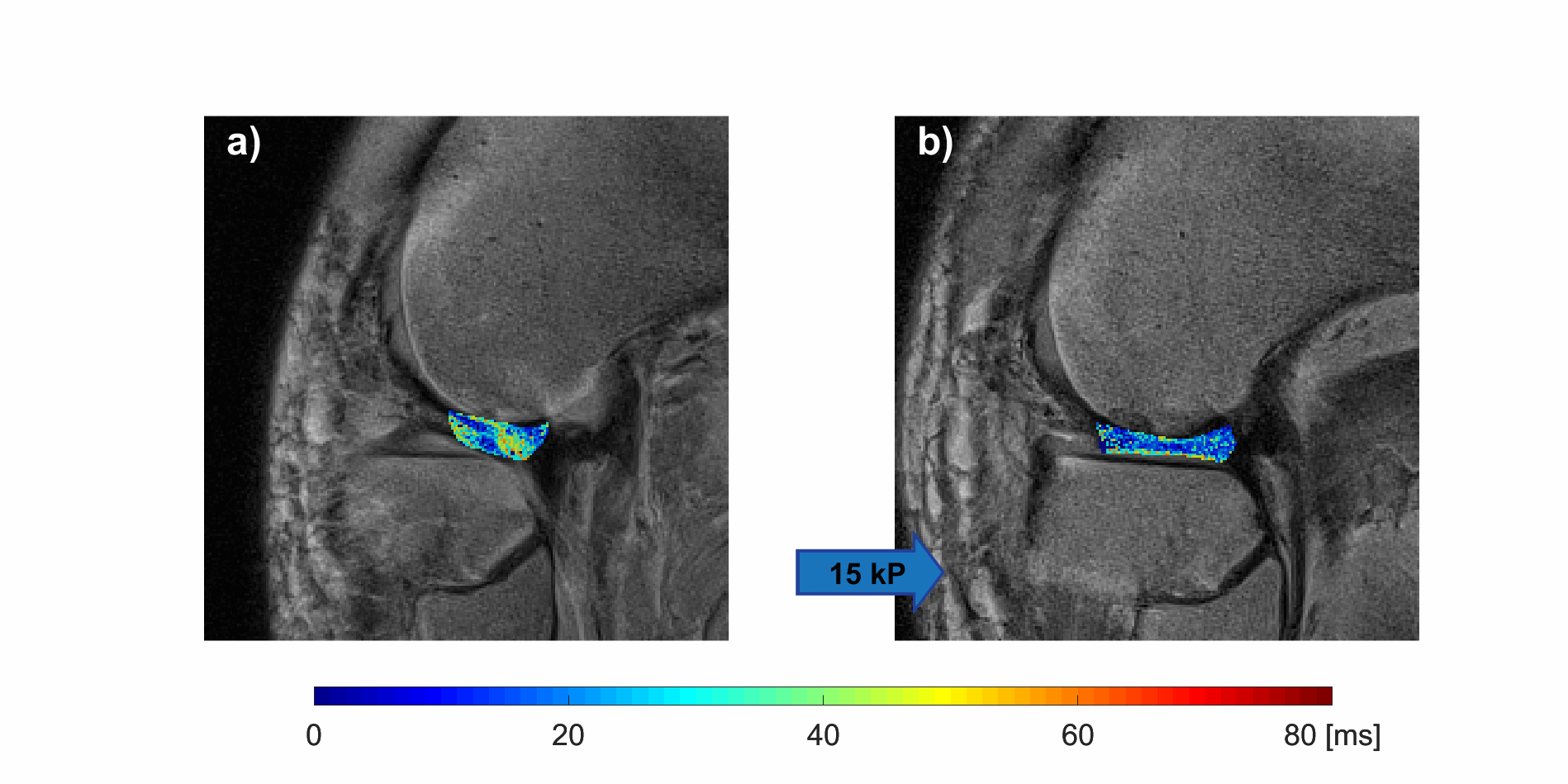

Supplement: Supplementary file 1 [file diagnostics-11-01790-s001.zip › diagnostics-1356909-supplementary.gif]
